# Identification of molecularly unique tumor-associated mesenchymal stromal cells in breast cancer patients

**DOI:** 10.1371/journal.pone.0282473

**Published:** 2023-03-20

**Authors:** Jonathan A. R. Gordon, Mark F. Evans, Prachi N. Ghule, Kyra Lee, Pamela Vacek, Brian L. Sprague, Donald L. Weaver, Gary S. Stein, Janet L. Stein

**Affiliations:** 1 Department of Biochemistry, Larner College of Medicine at the University of Vermont, Burlington, VT, United States of America; 2 Department of Pathology and Laboratory Medicine, Larner College of Medicine at the University of Vermont, Burlington, VT, United States of America; 3 Department of Surgery, Larner College of Medicine at the University of Vermont, Burlington, VT, United States of America; Università degli Studi della Campania, ITALY

## Abstract

The tumor microenvironment is a complex mixture of cell types that bi-directionally interact and influence tumor initiation, progression, recurrence, and patient survival. Mesenchymal stromal cells (MSCs) of the tumor microenvironment engage in crosstalk with cancer cells to mediate epigenetic control of gene expression. We identified CD90+ MSCs residing in the tumor microenvironment of patients with invasive breast cancer that exhibit a unique gene expression signature. Single-cell transcriptional analysis of these MSCs in tumor-associated stroma identified a distinct subpopulation characterized by increased expression of genes functionally related to extracellular matrix signaling. Blocking the TGFβ pathway reveals that these cells directly contribute to cancer cell proliferation. Our findings provide novel insight into communication between breast cancer cells and MSCs that are consistent with an epithelial to mesenchymal transition and acquisition of competency for compromised control of proliferation, mobility, motility, and phenotype.

## Introduction

Mesenchymal stromal/stem-like cells (MSC) are a heterogeneous population of cells that reside primarily in bone marrow and perivascular regions, but can also be found in limited quantities in adipose, muscle and other tissues, as well as circulating in peripheral blood [1]. The canonical role of MSCs is to regenerate and repair adult tissues through their multipotent capacity to differentiate into osteoblasts, adipocytes, chondrocytes, myoblasts, and in therapeutic applications, into cardiac myocytes and neurons [2]. Even though these cells provide a vital function, the steady state amount of MSCs in the bone marrow is relatively low compared to mononuclear and hematopoietic cells, ranging from 0.1–1 MSC per 10,000 cells [3]. In this capacity, MSCs are vital in their distinct ability to repair damaged tissues. These cells also have other regulatory properties including modulation of immune response [4], stimulation of capillary formation [5], regulation of hematopoietic stem cells [6], paracrine signaling through cytokine production [7, 8], as well as modulation of cancer cell activity [9–13].

MSCs are highly heterogeneous populations that have phenotypic variations based on the tissue of origin. However these cells do share some common basic molecular properties such as simultaneous expression of the surface markers CD73, CD90 and CD105 and the intrinsic property to differentiate in vitro into osteogenic, adipogenic and chondrogenic lineages. In addition to these requisite MSC characteristics, discrete populations of MSCs exhibit distinct molecular and phenotypic properties that may contribute to unique biological functions. MSCs isolated from embryonic umbilical cord have been demonstrated to express higher levels of specific cytokines including TGFβ (transforming growth factor-β) whereas adipose-derived MSCs express higher levels of VEGF-α (vascular endothelial growth factor-α) and EGF (epidermal growth factor) suggesting specific immunomodulatory and angiogenic potential among populations and/or individual MSCs. Additionally, subendothelial populations of MSCs characterized by the expression of CD146 and Angiopoietin-1 were shown to regenerate complete hematopoietic stem cell niches, which functionally support hematopoietic tissue [14, 15]. Compounding the functional complexity of these diverse populations and characteristics inherent to MSCs, different organs provide tissue-specific environments and may further give rise to MSCs with unique molecular and phenotypic characteristics.

Many properties of unique MSC populations can be induced by the cellular environment. MSC properties can be altered by routine processing, expansion in serum-free media, culture on surfaces of varying rigidity, serial passaging, and in vitro differentiation [16, 17]. Within a cellular context, subtle alterations in the cellular microenvironment including changes in pH, oxygen availability, ion gradients, extracellular matrix or cellular attachment substrates, can influence the growth and clonogenic heterogeneity of cells, resulting in distinct MSC subpopulations [18]. In contrast to tissue-resident MSCs, circulating MSCs, also called peripheral blood-derived MSCs (PB-MSCs), can be described as fibroblast-like cells that may generate colony forming units of cells with mesenchymal properties [19]. Similar to their bone marrow counterparts, circulating MSCs are very rare, and in normal individuals the frequency is as low as 1 MSC in 10^8^ peripheral blood mononucleocytes [20]. However, the relative frequency of PB-MSCs can be greatly increased in pathological conditions, such as osteosarcomas and other cancers, acute injury, multiple sclerosis and others [21, 22].

The tumor microenvironment of a solid tumor is a complex system of cellular and extracellular matrix interactions with a heterogeneous population of cancer cells with distinct phenotypical properties (differentiated, plastic or cancer stem-like cells). Cells that contribute to this tumor environment can be differentially organized into distinct cell types and biological roles, including immune cells establishing a modular immune status, vascular and hematopoietic cells promoting tumor angiogenesis and blood vessel formation, and epithelial and fibroblastic cells depositing the extracellular matrix, signaling molecules and cytokines. Within this tumor microenvironment, MSCs can play a distinct role; however that role remains to be definitively resolved. Studies have demonstrated that MSCs originating from bone marrow or adipose tissue can negatively affect tumor proliferation and promote apoptosis [23, 24], whereas other investigations have suggested that MSCs may aid tumor progression through promoting epithelial-mesenchymal transition (EMT) and increasing the proportion of cancer stem cells or increasing vascularization of tumors [25–27]. Due to the invasive growth of cancer cells, production of inflammatory signaling molecules and localized tissue damage, the tumor environment is generally pro-inflammatory, resulting in the recruitment of immune response cells, and can induce functional changes in these cells, including the conversion of monocytes/macrophages to tumor-associated macrophages (TAMs) [24]. Perhaps due to their regenerative potential, MSCs may also be recruited to sites of cancer cell-induced lesions to promote tissue repair and interact with cells within the tumor microenvironment through cell-to-cell contacts or through the expression and secretion of inflammatory cytokines, chemokines, growth factors, and other factors. It is important to note that the direct and/or indirect interactions of MSCs with cancer cells have been shown to influence changes in the tumor; however reciprocal interactions may promote differentiation or clonal selection of distinct MSC subpopulations with specialized functions that are unique to the tumor microenvironment [1, 28]. This phenomenon is demonstrated by the prevalence of cancer-associated fibroblasts (CAFs), which are derived from MSC progenitors that differentiate to adopt a fibroblastic, spindle-like morphology and are endowed with additional capabilities to enhance cancer stemness, leading to treatment resistance and tumor aggressiveness [29–32].

In this study, we have identified a population of CD90+ MSCs residing in the tumor microenvironment of patients with invasive breast cancer. These cells isolated from patients demonstrate a unique gene expression signature compared to normal individuals. The population was analyzed by single cell transcriptional analysis to identify a distinct subpopulation characterized by increased expression of genes related to extracellular signaling mediators. Blocking one of these pathways (TGFβ1) established that these cells directly contribute to cancer cell proliferation and expression of genes linked to epithelial to mesenchymal transition. Our results indicate that this cell population in patients may be a viable prognostic indicator, or by inhibiting function, a potential therapeutic target.

## Results

### Stroma from breast cancer patients contain CD90+ MSCs

**Fig 1:**
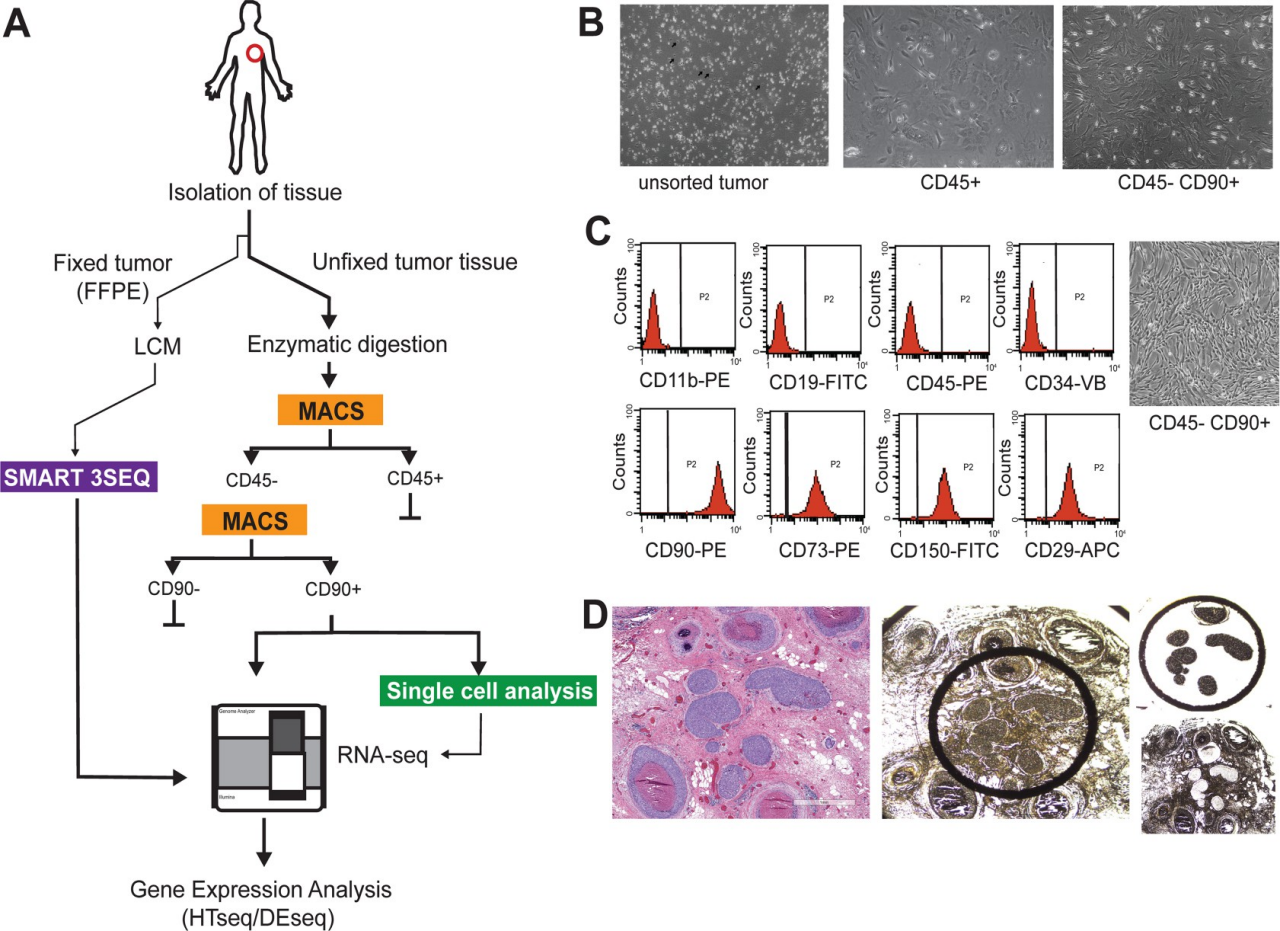
Schematic of sample acquisition for analysis. A) CD90 positive mesenchymal stromal cells isolated from breast cancer patients express a unique gene expression profile indicative of increased cell to cell signaling. MSCs were isolated from fresh stromal tissue adjacent to an invasive lesion (tumor-associated) or distal to the tumor site (patient-normal). B) Microscopy images demonstrating phenotypic characteristics of CD45+ or CD45-/CD90+ cells isolated by magnetic-activated cell sorting (MACS). C) CD90+ cells were subjected to FACS for phenotypic mesenchymal associated cell surface markers. D) Microscopy images demonstrating tumor tissues used for FFPE gene expression analysis. Hematoxylin and Eosin staining of tissue sections used for analysis (left panel) and images of tumor sections before and after Laser Capture microdissection (LCM) (right panels).

For this study we utilized a multifaceted sampling approach to evaluate gene expression signatures related to MSCs residing in the breast stroma of patients with screen detected invasive breast cancer, symptom detected breast cancer and DCIS (Fig 1). A small amount of breast tissue (~4 mm^3^, 0.019–0.025 g) was sampled from 15 individual patients undergoing resection of screen detected invasive breast tumors. From each patient two individual regions were sampled by biopsy punch, one region distal from the pathologically identified tumor (referred to as “patient normal stroma” (PNS)) and one region directly adjacent and/or containing tumor tissue (referred to as “patient tumor stroma” (PTS)). After enzymatic digestion, cells were isolated by sequential magnetic-activated cell sorting (MACS) with specific antibodies to lineage-related cell surface receptors to separate CD31+/CD45+ cells and subsequently in a separate isolation, CD90+ cells (Fig 1B). The majority of cells recovered were CD45+, with a small number (1.1 x 10^4^−3.4 x 10^5^, 0.1–0.3%) of CD90+ cells recovered for each patient (S1A Fig). Microscopic examination of cells demonstrated that the morphology of CD45+ cells was consistent with a mixture of hematopoetic-and epithelial-related cell types, whereas CD90+ displayed a distinct mesenchymal/fibroblastic morphology (Fig 1B). To further evaluate this CD90+ population, we subjected these cells to flow cytometry analysis using a panel of cell surface markers negatively (CD11b/CD19/CD45) or positively (CD73/CD90/CD105) associated with mesenchymal cell lineages. From this analysis the majority of cells (98.9%) isolated by the MACS procedure were shown to express mesenchymal stromal cell properties (Fig 1C). Additionally we sought to determine if we could identify MSC gene signatures within the breast cancer resident stroma using archival fixed tissue from a cohort of 20 patients with invasive breast cancer and 20 patients with DCIS (S1 Table). Formalin-fixed paraffin embedded (FFPE) sections were subjected to laser-capture microdissection to evaluate gene expression profiles in both tumor and tumor-associated stromal regions (Fig 1D).

### Patient-specific MSCs display a distinct molecular signature compared to normal MSCs

**Fig 2:**
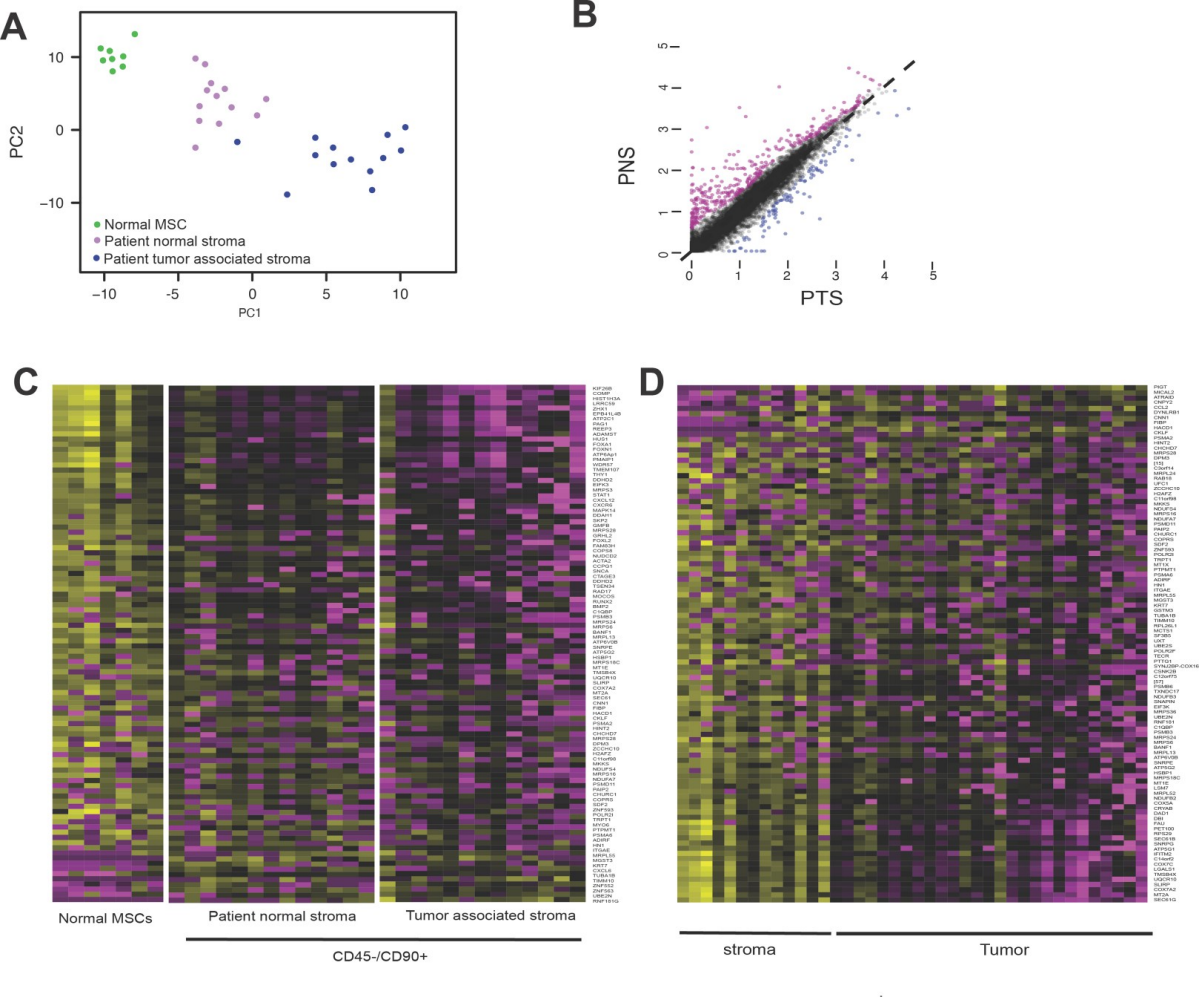
Gene expression analysis of patient-derived stromal and tumor samples. A) Principal component analysis (PCA) of gene expression profiles from MSCs isolated from patients. PCA of 35,264 transcripts expressed in MSCs isolated from unaffected young individuals (normal), unaffected tissue from breast cancer patients (PNS), or patient tumor stroma associated sites (PTS). Gene signatures in CD90+ MSCs from breast cancer (BC) patients are sufficient to distinguish between breast cancer patients and healthy individuals as well as tumor associated site and unaffected site. B) Scatterplot of gene expression demonstrating that tumor-associated MSCs (PTS) demonstrate gene expression changes compared to a distal site (PNS). C) Heatmap demonstrating trends in expression of 133 genes that showed significant variation between CD45-/CD90+ cells isolated from normal, patient normal stroma and patient tumor stroma. Gene signatures from MSCs were subjected to hierarchal clustering analysis but could not define cancer subtypes, but could define MSCs from normal or adjacent to tumor tissue. D) Heatmap demonstrating trends in expression of genes in Laser Capture Microdissection (LCM) of stromal and tumor samples derived from FFPE tissue and processed by SMART 3SEQ. Gene signatures from samples subjected to hierarchal clustering analysis and distinct gene expression signatures relating to MSC genes could differentiate stroma versus tumor tissues.

To evaluate gene expression profiles of patient-derived MSCs, we generated whole transcriptome libraries from patient-derived MSCs from normal stroma (PNS, n = 13), tumor-associated stroma (PTS, n = 13) or normal MSCs derived from normal bone marrow donors (n = 7). After QC of NGS sequencing data (S1 Table), differentially expressed genes (DEGs) were evaluated by differential expression analysis (DESeq2) and subjected to principal component analysis (PCA) (Fig 2A). This strategy provided 3 distinct sample groupings correlating to normal, PNS or PTS origin of MSC cells (Fig 2A). Based on the thresholds set for fold-change and *p* value, and correcting for interpatient variation, comparing the differentially expressed genes (DEGs) for transcriptomes of PNS versus PTS demonstrated that 485 genes were differentially expressed between the two patient-derived MSC groups, with 119 downregulated genes and 366 upregulated genes in PNS compared to PTS (Fig 2B). This result indicates that MSCs derived from tissue near but not adjacent to tumor demonstrated a distinct gene expression profile compared to MSCs directly adjacent or associated with breast cancer lesions. Similarly, evaluating differences in gene expression profiles from PNS, PTS and normal MSCs and after correcting for interpatient/donor variation, we observed 336 DEGs between patient-derived and normal donor groups, with 96 genes downregulated in PNS/PTS and 240 genes upregulated compared to normal donor-derived MSCs (S2B Table). From these DEG expression profiles we selected a subset of 103 genes that had the highest fold-change or lowest associated p-value to use as a discriminatory gene expression module to identify patient-related MSCs in other patient samples (Fig 2C). Using this gene expression module, we examined the expression of these genes in FFPE-LCM tumors and associated stroma (Fig 1D) from a cohort of patients with invasive breast cancers of varying grades and pathological status (S1 Table). This small subset of genes (103) demonstrated differential expression between the tumor and stromal compartments of the tumor (Fig 2D), suggesting that the DEGs from patient-derived MSCs residing in the stromal compartment are distinct from DEG signatures provided from the local tumor cells.

### Single cell transcriptomics reveal a distinct population of breast cancer associated MSCs

**Fig 3:**
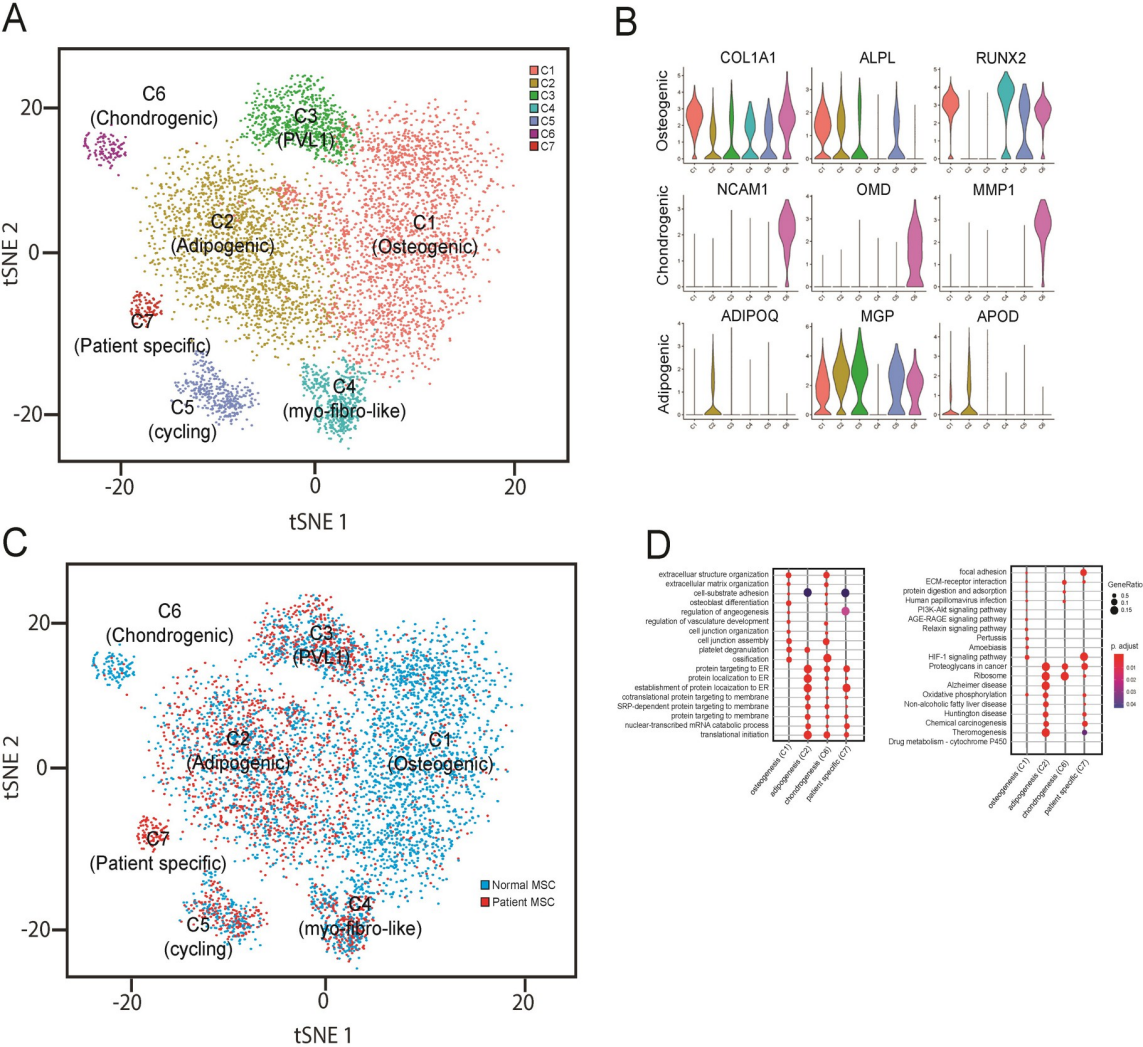
Single cell analysis of patient-derived MSCs. A) CD90+ cells were isolated from normal donors or patients with invasive breast cancer and subjected to single cell analysis. Gene expression profiles from single cells were clustered using tSNE, and 7 distinct cell clusters were observed. B) Candidate gene expression profiles were used to functionally characterize MSCs into 3 main subclasses (osteogenic, chondrogenic or adipogenic). C) Comparison of cells derived from healthy donors or breast cancer patients demonstrated proportional changes in number of cells contributing to specific clusters. D) Ontology categories associated with single cell populations.

**Fig 4:**
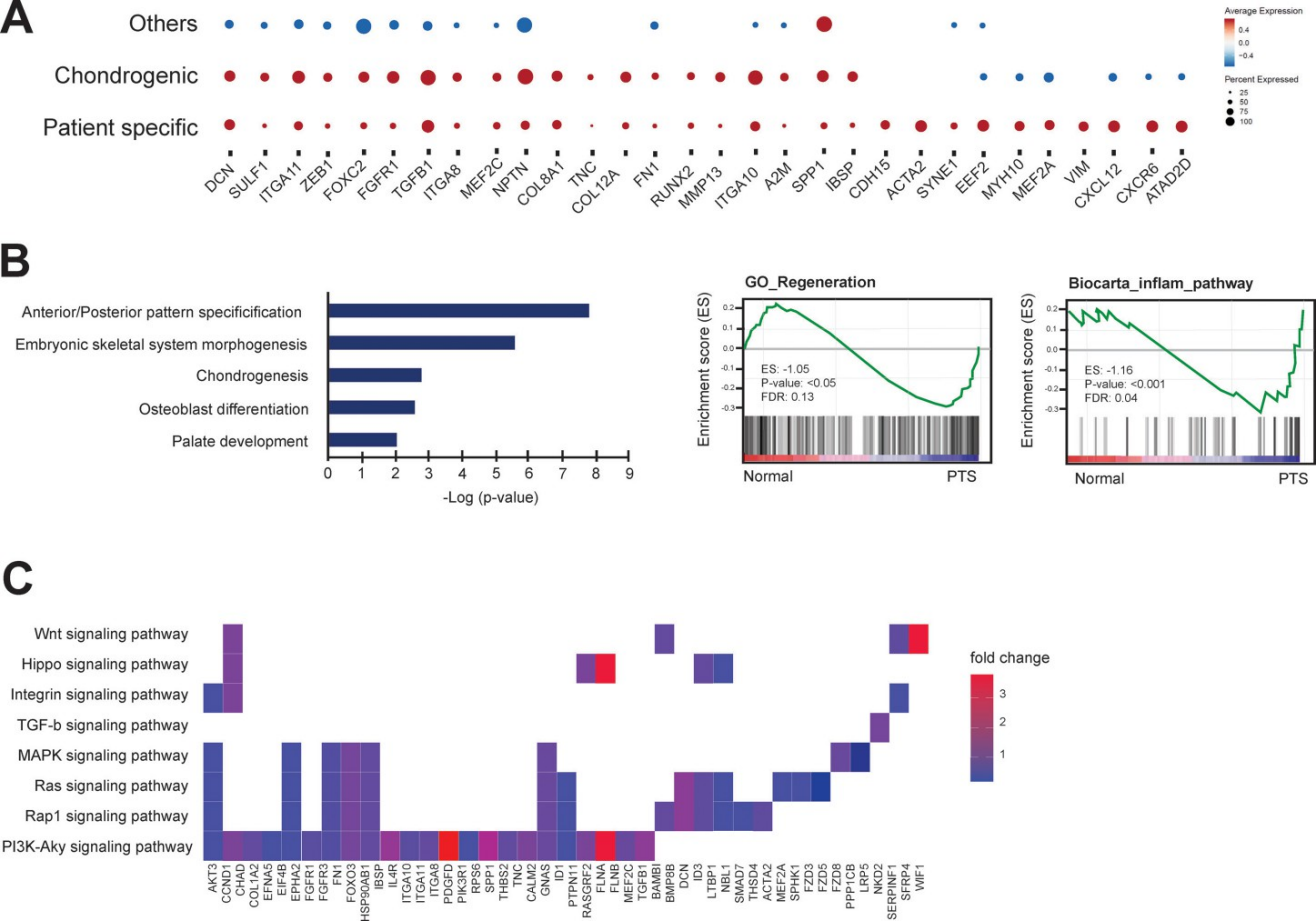
Pathway and ontology analyses reveal that patient-derived MSCs demonstrate unique properties. A) Dot plot of proportion of cells in the respective cell clusters (Other, Chondrogenic, Patient-specific) expressing each gene (dot size), and average expression (color scale). B) Ontology and GSEA enrichment plots demonstrating category association of gene signatures expressed by patient-specific MSCs. C) Gene expression pattern in enriched pathways. Squares show enriched DEGs in the corresponding terms (rows). Color indicates the expression value of the DEGs (average logFC).

Given the relatively diverse function of MSCs in tissue homeostasis and the heterogeneity of MSCs derived from the bone marrow compartment, we sought to examine if there were distinct populations of cells within the CD90+ MSC populations derived from patients. We processed 3 patient-derived samples and 4 normal (donor-derived) CD90+ MSC samples by MACS, and single cell suspensions were generated using the 10x Genomics Chromium platform to evaluate individual cell gene expression profiles. For each patient or donor an average of 3108 (± 456) cells/sample for a total of 23,633 cells were evaluated with a median of 2557 genes/cell and a median of 12215 UMIs/cell (Fig 3A and S3 Table). Unsupervised clustering of the gene expression profiles using Seurat and mapping using tSNE revealed 7 distinct groups (C1-C7) of mesenchymal lineage cells (Fig 3A). To identify properties of the cells of each cluster, we used gene expression modules related to specific MSC-related lineages including osteoblasts, adipocytes, and chondrocytes to define phenotypic identities of individual populations. Cells with high expression of COL1A1, ALPL, RUNX2 among 10 other osteoblast-related genes (e.g., OPG, COL1A2, SP7) were classified as osteoprogenitors (C1) whereas cells expressing 6 adipocyte-related genes (e.g., ADIPOQ, MGP, APOD) were classified as adipoprogenitors (C2) and cells expressing chondrocyte-related genes (e.g., NCAM1, OMD, MMP1) were identified as chondroprogenitors (C6) (Fig 3B). Gene expression profiles for the C1 and C2 classes made up the majority of examined cells, with adipoprogenitors being the most abundant (42.3%) followed by osteoprogenitors (41.3%) with the remaining clusters contributing a small proportion of the total (16.4%). In cluster C3 the majority of the variation in gene expression was genes involved in cell cycle regulation. In cluster C3, most of the cells showed increased expression of G2/M cell cycle-related genes (e.g., CDC5A, MELK, RACGAP1, PRC1, and HMGB1) suggesting the major demarcation of this cluster is cell cycle stage and not unique differentiation or lineage-related progenitor properties. Interestingly, when cells were displayed as patient-derived or donor-derived, the proportion of cells in each cluster varied dramatically (Fig 3C). Patient-derived MSC clusters had a significant reduction in the number and proportion (6.8%) of cells with osteoblast-related gene expression (C1), and a significant increase in the number (67.8%) of cells expressing adipocyte-related genes (C2), and an almost complete absence of cells (0.6%) with chondroprogenitor characteristics. Strikingly, cluster C7 was restricted to only patient tumors and did not have any contribution from normal donor-derived MSCs (0/16328 cells). The gene expression signature of this unique patient-specific population of CD90+ MSCs was subjected to pathway analysis compared to cells of the 3 defined and classified lineages (osteo/adipo/chondro progenitors) (Fig 3D). The gene signature of the C7 population was associated with pathways and genes involved in cell matrix adhesion (e.g., COL8A1, COL10A1 COL12A1, ITGA11) and genes downstream of various signaling pathways including TGFβ. From this pathway analysis, it is clear that this population is molecularly and presumably phenotypically distinct from the other CD90+ MSC cell clusters identified. To understand how the patient-specific MSCs relate to other populations of MSCs, we plotted expression patterns of spatially localized genes among our defined clusters and specifically versus chondrogenic-related MSCs (Fig 4A). First, some genes (DCN, SULF1, ITGA11, ZEB1, FOXC2, FGFR1) were elevated in both C6 (chondrogenic) and C7 (patient-specific) populations compared to all other clusters. Specific genes related to chondrogenesis (COL8A1, TNC, COL12A1, FN1, RUNX2, MMP13, ITGA10, IBSP) were elevated in a percentage of chondrogenic and patient-specific MSCs compared to other MSCs. In addition, several cytokine signaling and/or myogenesis genes (CDH15, ACTA2, SYNE1, EEF2, MYH10, MEF2A, VIM, CXCL12, CXCR6, ATAD2D) were expressed in a substantial proportion of patient-derived cells and were elevated compared to all other populations (including chondrogenic) (Fig 4A). From the GSEA and pathway analysis it was also evident that this population of MSCs expressed gene signatures that were significantly associated with other signaling pathways including WNT, Hippo, MAPK and PI3K-AKT (Fig 4B and 4C).

### Single cell analysis of breast cancer tumors reveals that MSC-like cells are the major source of collagen gene expression in bulk tumor samples

**Fig 5:**
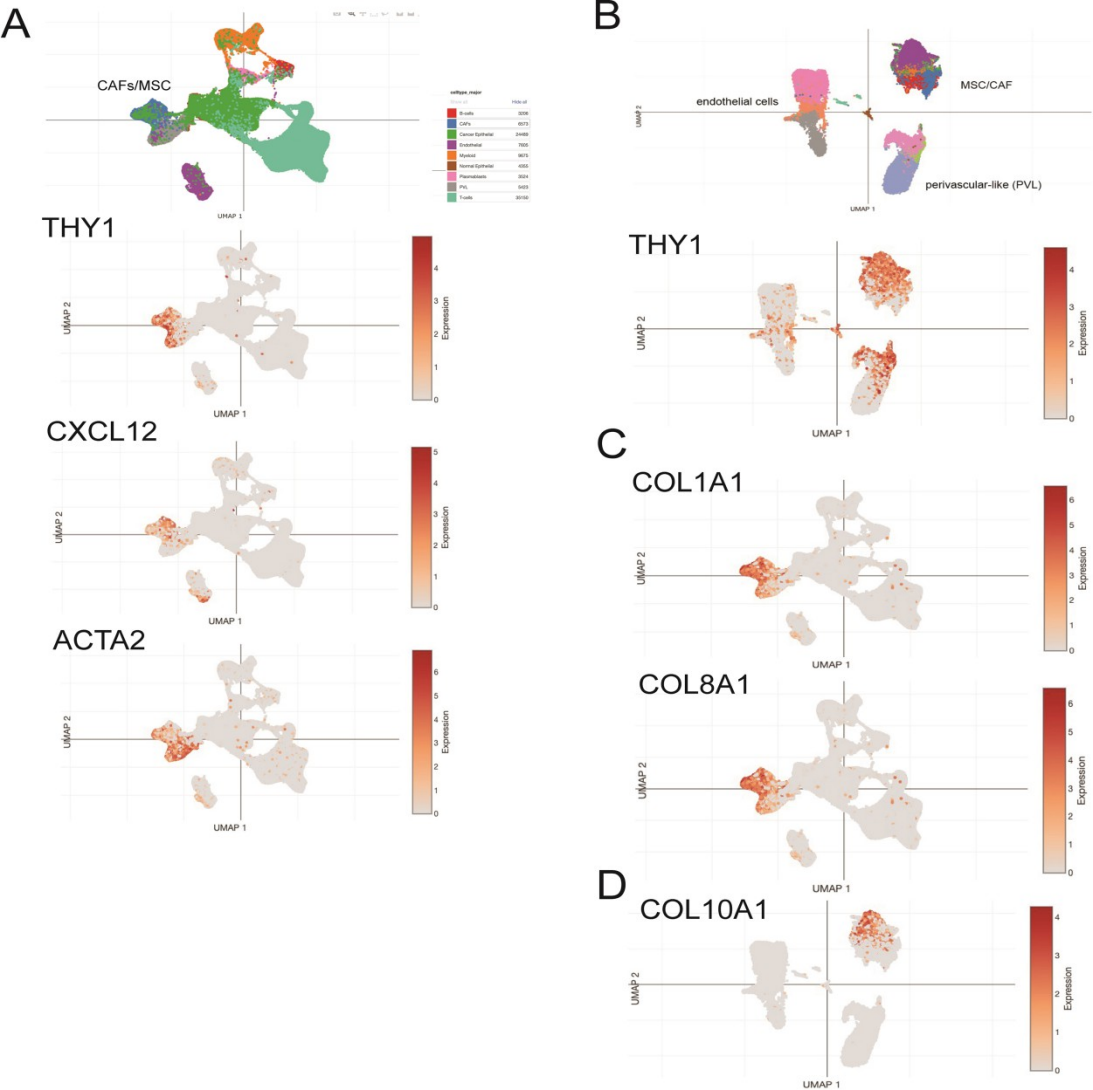
MSC-related gene expression can be observed in whole tumor single cell RNAseq. UMAP visualization of 130,246 cells analyzed by scRNA-seq and integrated across 26 primary breast tumors (from Wu et al [34]). Clusters were annotated for their cell types as predicted using canonical markers for epithelial cells (*EPCAM*), proliferating cells (*MKI67*), T cells (*CD3D)*, myeloid cells (CD68), B cells (*MS4A1*), plasmablasts (*JCHAIN*), endothelial cells (*PECAM1)* and mesenchymal cells (fibroblasts/perivascular-like cells; *PDGFRB)* and gene signature-based annotation. A) Non-tumor MSC, perivascular and endothelial cells cluster on left side of plot demonstrated the majority of THY1 (CD90), CXCL12 and ACTA2 expression in the whole tumor. B) UMAP visualization of reclustered mesenchymal cells, including CAFs (6,573 cells), PVL cells (5,423 cells), endothelial cells (7,899 cells), lymphatic endothelial cells (203 cells) and cycling PVL cells, demonstrating that the majority of CD90 (THY1) positive cells residing in the assigned MSC cluster. C) Feature plots of gene expression of COL1A1, COL8A1 in whole tumor UMAP demonstrating gene expression restricted to MSC-associated clusters, and D) MSC UMAP demonstrating COL10A1 gene expression restricted to MSC/CAFS.

We interrogated single cell data from a robust, publicly available dataset of spatially resolved human breast cancers [33] to ascertain if the observed gene signatures from our patient-derived CD90+ MSCs were unique to MSCs or could be observed in other cells composing the tumor microenvironment. We examined cells separated by gene expression profiles (by UMAP) allowing distinct lineage-specific cells to be spatially resolved. In cell clusters associated with MSCs or the progeny (i.e., cancer-associated fibroblasts, CAFs) in the whole tumor data, it was evident that MSC-related cells were the major source of THY1 (CD90), CXCL12 and ACTA2 expression in breast cancer patient samples (Fig 5A). Stromal cell populations from whole tissue plotted separately demonstrated that there is some THY1 expression in perivascular-like cells. The overwhelming expression of THY1 was in MSC-related populations (Fig 5B). Strikingly, examining the source of collagen gene expression (COL1A1, COL1A2, COL3A1, COL6A1, COL8A1, COL10A1) provided clarity that the majority of genes expressed in the tumor samples represent transcripts of the MSC-related component of the tissue microenvironment (Fig 5C). Comparing the gene signatures to the cancer-associated patient-specific MSCs isolated in our study provide evidence that the patient-derived cluster of cells (C7) had an increased level of COL10A1 expression; thus, these cells may be the source of COL10A1 expression in the tumor (Fig 5D). These data support our identification of a unique gene signature from patient-derived MSCs that may play a direct role in the tumor microenvironment by contributing a distinct complement of extracellular matrix molecules and signaling components.

### Inhibition of TGFβ pathways in breast cancer associated MSCs results in alteration of survival pathways in cancer cells

**Fig 6:**
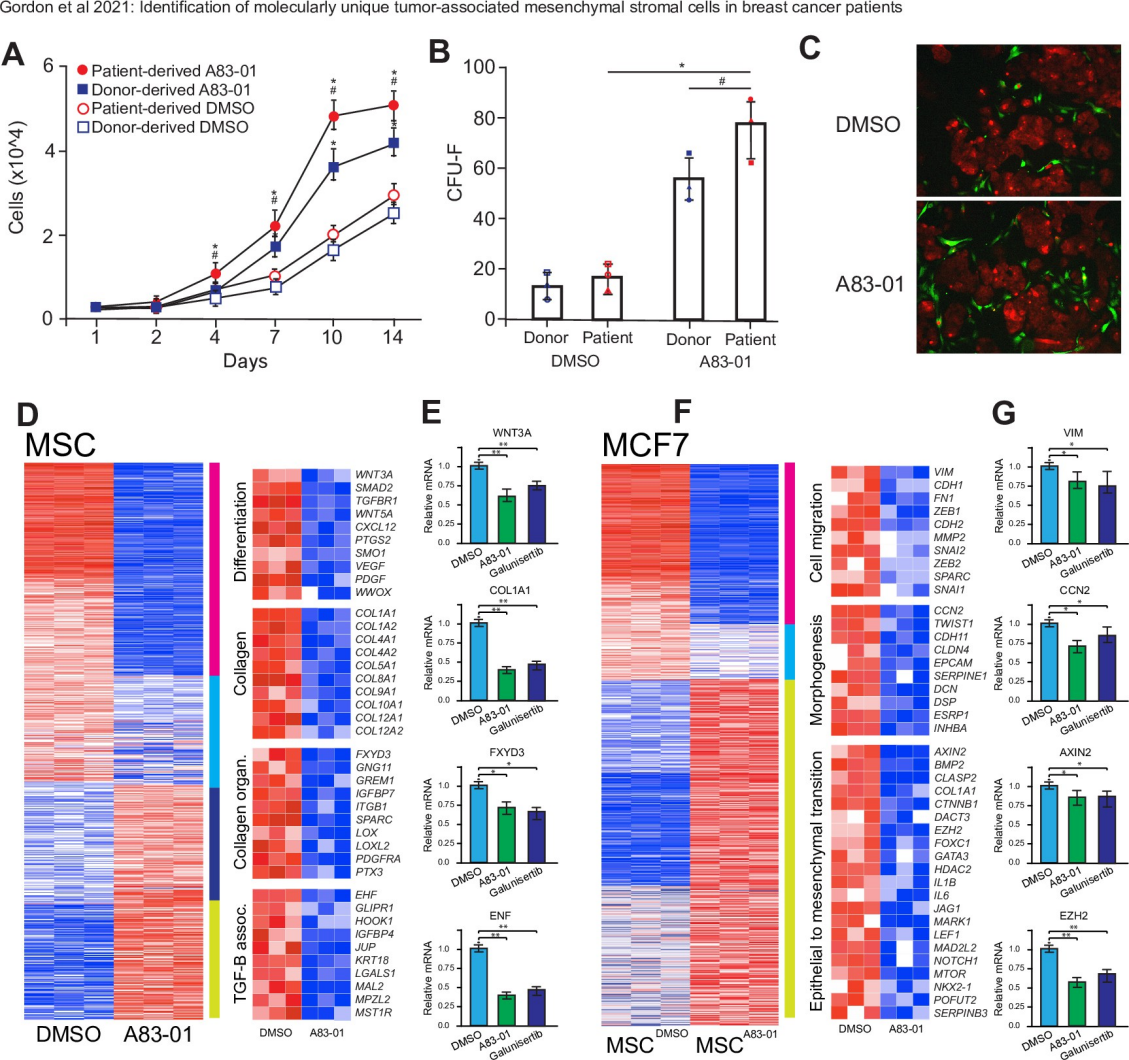
Treatment of MSCs with TGFβ inhibitor affects interactions with cancer cells. A) Cell proliferation assay of patient-derived or donor-derived MSCs treated with TGFβ-R1 inhibitors or control (DMSO). Points represent means with SEM. Significance was calculated by two-way repeated measures ANOVA using Tukey post hoc test and highlighted where p <0.05 (* A83-01 vs DMSO, ** Patient vs Donor). B) Colony formation assay measuring the number of colonies (CFU-F) formed after 7 day pretreatment with A83-01 or DMSO. Points represent individual patients or donors. Significance is highlighted where p <0.05 (* A83-01 vs DMSO, ** Patient vs Donor). C) Co-cultures of patient-derived MSCs with the estrogen receptor positive MCF7 breast cancer cell line in cells pretreated with Control (DMSO, left) or TGFβ R1 inhibitor (A8301, right). Scale bars represent 200 μm. D) Heatmap demonstrating differential gene expression in patient-derived MSCs treated with DMSO or A83-01 and E) associated relative gene expression (qPCR) of cells treated with DMSO, A-83-01 or Galunisertib. Significance was calculated Student’s t-test and highlighted where p <0.05 (*) or p <0.01 (**). F) Heatmap demonstrating differential gene expression in MCF7 cells co-cultured with patient-derived MSCs treated with DMSO or A83-01 and 01 and G) associated relative gene expression (qPCR) of cells treated with DMSO, A-83-01 or Galunisertib. Significance was calculated Student’s t-test and highlighted where p <0.05 (*) or p <0.01 (**).

Due to the upregulation of TGFβ related genes in patient-specific MSCs, we targeted this pathway for inhibition through treatment with an agonist for the activin receptor (A83-01) that selectively inhibits TGFβ-related receptors ALK4, ALK5, and ALK7 and the phosphorylation of their downstream targets SMAD2 and SMAD3 [35]. To evaluate the effect of sustained TGFβ-R inhibition on cell growth and proliferation, cells isolated from 3 individual patients were seeded at 5000 cells/cm2 in media supplemented with either a selective inhibitor of TGF-βRI, ALK4 and ALK7 (A83-01) or vehicle (DMSO). The medium was refreshed every 48 h and cumulative cell populations were calculated at each time point (culture days 1,2,4,7,10,14). Treatment with A83-01 from culture initiation progressively and significantly (p < 0.05) conferred a proliferation advantage on patient-derived MSCs compared to DMSO treated controls (Fig 6A). Cells isolated from normal donors demonstrated a similar increase in proliferation, but at a significantly (p = 0.034) reduced rate compared to patient-derived cells. Patient-derived MSCs initially cultured with A83-01 or control (DMSO) for 1 passage were seeded at a low density (50 cells/cm2) to allow colony formation for a further 14 days. A83-01 treatment significantly increased the number of fibroblast colony forming units (CFU-F) of patient-derived cultures compared to DMSO control (p = 0.028) and normal donor-derived MSCs (p = 0.026) (Fig 6B).

The estrogen receptor-positive, breast cancer cell line MCF7 has been previously used to demonstrate a link between MSCs, TGFβ—related signaling and the promotion and maintenance of prometastatic epithelial to mesenchymal transition [36]. We were interested in how patient-derived MSCs may interact to promote cancer-related phenotypes. To assess the effect of TGFβ-Receptor inhibition on interaction with cancer cells, patient-derived MSCs pretreated with A83-01 or DMSO and CellTracker Green CMFDA Dye were co-cultured with MCF7 cells stably expressing mCherry (Fig 6C). In co-cultures seeded with DMSO-treated MSCs, MCF7 cells formed tightly organized colonies with fibroblastic morphology that interacted with MSCs on the periphery and within the MCF7 colonies themselves, as evidenced by yellow co-labeling (Fig 6C upper panel). In contrast, in coculture experiments using A83-01-treated MSCs, there was a notable difference in MCF7 morphology, colony formation and apparent MSC interaction (Fig 6C lower panel). Although there were observed interactions (through colocalization labelling) with A83-01-treated, patient-derived MSCs and MCF7 cells, these events were usually isolated (i.e., not in colonies) (Fig 6C lower panel) and were strikingly different than control culture with MCF7 cells showing less colony-forming characteristics and less cell to cell contact. Co-cultures of patient-derived MSCs and MCF7 cells treated with DMSO or A83-01 were then separated by FACS and subjected to gene expression analysis by RNAseq. Following batch and statistical correction for multiple-testing, 873 genes were differentially expressed upon A83-01 treatment compared to DMSO-treated controls (Fig 6D). Of these genes, 321 were highly down-regulated (magenta cluster) and 370 were up-regulated in DMSO-treated cells. Gene ontology (GO) and GSEA analysis revealed that genes down-regulated upon A83-01 treatment are enriched in similar biological processes that distinguish patient-specific MSCs from other MSC clusters, including regulation of cell differentiation (p = 7.8x10^-8^), extracellular matrix (collagen) (p = 4.5x10^-10^), collagen fibril organization (p = 4.6x10^-6^) and TGFβ associated pathways (p = 5.8x10^-8^). Notably, in these categories several of the significantly downregulated genes upon A83-01 treatment encode extracellular matrix (ECM) components, including various collagen subunits (e.g., COL1A1), and are genes that define patient-specific MSC populations versus normal MSCs. To confirm that genes that were identified by A83-01 inhibition were specific to the inhibition of TGFβ, we also treated MSC with the clinically approved TGFβ inhibitor Galunisertib (LY2157299) and evaluated the several genes (WNT3A, COL1A1, FXYD3, ENF) in each category for relative expression by qPCR (Fig 6E). We also evaluated gene expression profiles of MCF7 cells co-cultured with MSCs pretreated with DMSO or A83-01 (Fig 6F). In total 873 genes were differentially expressed by co-culture with patient-derived MSCs that had been treated with A83-01 (versus DMSO) with an upregulation of 335 genes in total in DMSO controls (compared to TGFβ signaling inhibited (i.e., A83-01-treated MSCs). Breast cancer cells interacting with patient-specific MSCs treated with DMSO showed an increase in genes associated with cell migration (e.g., VIM, CDH1, FN1, ZEB1, MMP2, SPARC), tissue morphogenesis (e.g., CCN2, TWIST1, CDH11, DCN, ESRP1) and epithelial to mesenchymal transition (e.g., AXIN2, BMP2, COL1A1, CTNNB1, EZH2, FOXC1, JAG1, MARK1, LEF1, NOTCH1), compared to MCF7 cells co-cultured with patient-derived MSCs treated with TGFβ inhibitor (Fig 6F). In addition, gene expression changes in MCF7 cells cocultured with MSCs treated with clinically approved TGFβ inhibitor Galunisertib were similar to A83-01 for representative genes in each pathway/ontology category (e.g. VIM, CCN2, AXIN2, EZH2) (Fig 6G). Taken together, these results suggest that activation of TGFβ pathway effectors in MSCs influence several pathways related to cancer progression in interacting breast cancer cells.

## Discussion

The tumor microenvironment plays a distinct role in all aspects of tumorigenesis, including the establishment of oncogenic character, maintenance and growth of the malignant tumor and malignant/metastatic progression. It is well established that within the tumor microenvironment cells of the immune system, such as macrophages, neutrophils, mast cells, myeloid-derived suppressor cells (MDSCs), dendritic cells (DCs), and natural killer (NK) cells and those of adaptive immunity (T and B lymphocytes), play a complex role in the tumor niche. Normally, in cases of an invading pathogen, these cells would act to initiate an inflammatory response; however in the tumor microenvironment, these cells are frequently dysregulated or functionally impaired, which in some cases can promote cancer progression through extracellular signaling effectors and recruit or modify cells in the surrounding environment [37]. A significant portion of the surrounding environment is composed of tumor stromal cells of mesenchymal origin (mesenchymal stromal cells, fibroblasts, endothelial cells, and pericytes). Interactions that occur between immune cells and cells of the tumor itself are highly complex, and involve numerous cytokines, chemokines, and mitogens that directly affect tumor progression and outcomes.

The role of MSCs in tumorigenesis is subject to discussion, as several studies have demonstrated that MSCs have a protective or tumor-suppressive role [38–41]. Others have demonstrated that MSCs may aid, promote and direct tumor formation in a wide range of primary tumors [42–46]. MSCs are pro-tumorigenic in epithelial cancers including breast, colon, lung, and prostate, as well as hematopoietic malignancies, such as multiple myeloma and leukemia [47–53]. The mechanistic role of MSCs in cancer pathogenesis is currently under intense scrutiny; several lines of evidence point to MSCs having distinct roles during tumor progression. MSCs provide immunosuppressive properties in the tumor locale by direct and/or indirect cell-to-cell communication with immune cells and/or cancer cells, ultimately to regulate the tumor microenvironment [54, 55]. The interaction of MSCs with immune cells may alter populations of immune cells in response to cancer-induced inflammation and temper immune reactions directed against malignant cells. It is evident that different immune environments may drive tumor heterogeneity as recruited B- and T-lymphocytes define three distinct immune states, characterized by histological and molecular features of immune escape [56] which may be driven in part, by MSCs interactions in this environment. A reciprocal interaction can influence the conversion of naïve MSCs to a pseudo-differentiated state that has distinct phenotypic properties.

We have identified a distinct population of MSCs from a native tumor microenvironment that shows gene expression signatures that suggest an increased potential for response to extracellular stimuli. This population of MSCs expresses genes associated with TGFβ, MAPK and WNT signaling, among others (Fig 4C), which suggests that these cells have been activated in response to effectors of these pathways or are primed to receive future signals. This activation or priming may be in response to inflammation from the tumor, thus eliciting a tissue regeneration response from resident or distal MSCs. Numerous studies have demonstrated that MSCs are mobilized in response to inflammation events and participate in wound healing or tissue regeneration responses [57]. The reactive stroma derived from MSCs has frequently been designated as cancer-associated fibroblasts (CAFs), and several studies have shown that resident MSCs differentiate into myofibroblast-like (myCAFs) cells in response to TGFβ-related signals [58–60]. There is a great deal of phenotypic overlap between the proposed functions of CAFs and CD45-/CD90+ cancer-associated MSCs but it would appear that MSCs, and specifically cancer-associated MSCs identified in this study, may be progenitors for canonical CAFs. It has been proposed that CAF subtypes (i.e., iCAFs and myCAFs) represent states of phenotypic plasticity with some degree of interconvertibility with indeterminate terminal differentiation states [58], suggesting that CAFs may in large part retain multipotent potential and overlap with cancer-associated MSCs. In addition, TGFβ-stimulated CAFs have been a suggested target to treat unresponsive tumors restoring trastuzumab anti-cancer efficiency through increased IL-2 production [61]. Overall, our study underscores the therapeutic potential of exploiting the tumor microenvironment to identify and overcome mechanisms of resistance to anti-cancer treatment. It remains to be determined if these activated cancer-associated MSCs are recruited to or reside in the breast tissue. The identified cells from patients with invasive breast lesions may play significant and unique roles as markers of an immune response in relation to the tumor lesion.

MSCs can promote tumor vascularization by excretion of pro-angiogenic cytokines. Angiogenesis is mediated by several cytokines, including vascular endothelial growth factor (VEGF)-A, fibroblast growth factor (FGF), hepatocyte growth factor (HGF), placental growth factor (PlGF), and matrix metalloproteinases (MMPs) [62, 63]. MSCs are a primary source of pro-angiogenic cytokines due to their role in normal tissue repair [64, 65]. There is some evidence suggesting that in certain conditions, MSCs can differentiate directly into endothelial cells [66] and may have capacity to generate *de novo* blood vessels in response to the paracrine expression of pro-angiogenic factors [67]. Other evidence indicates that cells closely related to CD90+ MSCs, like nestin-positive, CD105/CD31-positive mesodermal progenitor cells and CD146-positive, α-SMA/desmin/NG-2/PDGFR-α expressing pericytes, can act with MSCs to generate new blood vessels in numerous tissues [67]. While the direct roles for MSCs promoting neovascularization are not definitively established, it appears that the population of MSCs isolated from patients with invasive tumors express pro-angiogenic genes, including PDGF-a, VEGF, CXCL12/SDF-1 and FGF (Fig 6D). These results suggest that MSCs have a significant role in promoting recruitment of angiogenic cells and new blood vessel formation, similar to the roles of MSCs in promoting blood vessel formation in bone and other tissues [68–70].

Perhaps the most important role of MSCs in the tumor microenvironment is that MSCs can directly influence cancer cells through expression and secretion of chemokines, growth factors, metabolites and extracellular matrix proteins. Reciprocally, cancer cells may influence the differentiation or transformation of MSCs into functionally distinct cell types. One of the key gene expression modules we identified in cancer-associated MSCs is genes related to extracellular matrix, including numerous collagen genes, as well as noncollagenous ECM proteins. It is striking that in the whole tumor single cell data from Wu et al. [34] the overwhelming majority of collagen expression is contributed by cells of the MSC/CAF lineage as opposed to epithelial or other cell types (Fig 5C). This result suggests that the majority of new collagen synthesis and deposition in the tumor stroma is due to the actions of MSCs/CAFs. Collagen composition, orientation and structural assembly have been identified as prognostic indicators of progression in breast cancer and other solid tumor types [71–74], an important finding that highlights the transformative potential of tumor-associated MSCs in altering the tumor microenvironment and therefore contributing to or driving changes in the tumor itself.

Single cell analysis of patient-derived MSCs suggests that even though most MSCs express COL1A1, several collagen genes such as COL10A1 and COL8A1 are expressed predominantly by patient-specific MSCs (Fig 5C). COL10A1 is generally associated with hypertrophic chondrocytes, but recent studies have linked COL10A1 expression to progression and/or patient-survival in a wide range of tumors, including colorectal, lung, gastric and prostate [75–77]. In addition, meta-analysis of breast cancer gene expression data has suggested that COL10A1 might be a predictive biomarker for prognosis of breast cancer [78], although the exact role of increased COL10A1 expression in breast tumor progression is currently unknown. Collagen expression by MSCs would change the tumor stroma and confer increased matrix stiffness, driving the loss of polarity, increased proliferation and invasiveness of cancer cells [79]. Increasing stiffness of the surrounding ECM has been shown to induce EMT in breast cancer cells by promoting TWIST1 to translocate into the nucleus [80]. Additionally EMT in cancer cells has been linked to a number of signaling effectors including WNT5A [81].

In this study MSCs that were pretreated with a TGFβ inhibitor demonstrated a marked reduction in WNT5a and several collagen genes (e.g., COL1A1, COL4A1, COL10A1 (Fig 6D)). When these MSCs were co-cultured with a breast cancer cell model, there was a coincident reduction in EMT-associated genes (e.g., ZEB1/2, TWIST1, SNAIL, FOXC2, N-Cadherin (Fig 6E)). The role of TGFβ signaling in inducing EMT in cancer cells has been fairly well characterized [82–85]. Our results suggest that TGFβ signaling may be more important in driving pro-tumor effects from cancer-associated MSCs. Several potential anti-TGFβ inhibitors are currently under clinical development in phase I/II trials to treat primary tumors [86], but others have relatively poor success in clinical cancer treatment [87]. Patients with primary tumor itself may respond to anti-TGFβ therapies, but patients with cancer-associated MSCs may benefit from a prophylactic or targeted treatment with TGFβ inhibitor to revert activated tumor-associated MSCs to a naïve state. Our findings provide novel insight into communication between breast cancer cells and MSCs that are consistent with acquisition of competency for compromised control of proliferation, mobility, motility, and phenotype.

## Materials and methods

### Isolation of MSCs from breast cancer patients

Patient-derived MSCs were obtained from fresh breast tumor specimens from patients undergoing surgery at University of Vermont Medical Center. Tumor tissues were washed with PBS, cut into small pieces, and digested with 3 mg/mL collagenase I (Sigma) and 5 MU/mL of DNase I (Calbiochem) in PBS for 2 hours at 37°C. Cells were passed through a 70-μm strainer filter and negatively selected for CD45 expression using CD45 Microbeads (Miltenyi Biotec) and separated using magnetic cell separation LD columns (Miltenyi Biotec). The remaining cells were positively selected by treating with CD90 Microbeads and then selected using an MS column (Miltenyi Biotec). Cells were collected by centrifugation and then frozen (-150°C) in Bambanker until used directly for experiments. To confirm that isolated MSCs retained mesenchymal phenotype, cells were analyzed by flow cytometry to confirm the expression of CD29, CD90, CD44, and CD105 but not CD45, CD34, and CD11b at passage 2. All experiments were performed with subconfluent cells in the exponential phase of growth. Normal, donor-derived MSCs were obtained from fresh bone marrow samples from patients with Texas A&M Center for Regenerative Medicine Biobank. MSC when cultured, were maintained in ascorbic-acid free aMEM supplemented with l-glutamine and penicillin-streptomycin. MCF7 cells were purchased from ATCC (Manassas, VA) and maintained in DMEM-F12 complete media supplemented with 10% fetal bovine serum (FBS), MEM nonessential amino acids, gentamicin and 10μg/mL insulin in a 5% CO2 incubator at 37°C. Media was changed every 2–3 days and cells were passaged when 65–80% confluent. MCF7 cells and mCherry-expressing clonal MCF7 cells were subjected to STR analysis and karyotyping to determine cell identity and genomic drift.

All research performed in this study was approved with a waiver of consent by the University of Vermont Institutional Review Board (study 15–629) to obtain samples from the University of Vermont Cancer Center (UVMCC) BioBank (study M13-238). Samples from the UVMMC BioBank were obtained from excess clinical tissue collected with written consent.

### Flow cytometry for surface expression markers and intracellular signaling

MSCs and patient-derived MSCs were harvested with PBS-based cell dissociation buffer (Gibco; 1 × 105 cells per tube), washed, suspended in PBS with 5% BSA, and stained with marker-specific fluorochrome-conjugated antibodies for 30–60 min in the dark. Nonspecific background signals were measured by incubating separate tubes with appropriate isotype-matched non-specific antibodies. Cells were then resuspended in PBS + 5% BSA and filtered through a 40μm cell strainer (Flowmi) and sorted by a LSRII flow cytometer (BD Biosciences). Cell debris and clumps were electronically gated from analysis based on their forward and side light scatter parameters.

### Cell viability, proliferation assays and co-culture assays

Cell viability for MSCs and A83-01-treated MSCs was assessed by adding CellTiter-Blue reagent (Promega) according to the manufacturer’s instructions. After 4 hours of treatment, fluorescence (560Ex/590Em) was measured in a Victor X2 fluorescence plate reader (Perkin Elmer). Data were calculated in relative fluorescence units and converted to previously determined normalized fluorescence values per cell and displayed as cell number. For proliferation analysis, normal MSCs, patient-derived MSCs and/or treated MSCs were plated in triplicate in 96-well plates at a density of 500 cells per well. Cells were incubated at 37°C and at specified time points (1–14 days post-plating) cells were treated with viability stain and counted. For fibroblast colony forming unit (CFU-F) assays, MSCs, patient-specific MSC or A83-01-treated cells were resuspended and seeded at a density of 50 cells/cm^2^ into 12 well plates in triplicate. After 3 days of culture the number of fibroblast like colonies were imaged on a Zeiss AxioImager2 equipped with Hamamatsu CCD camera, and images were captured using Zeiss Zen2012 software. Image analyses and quantification were performed using ImageJ (https://imagej.nih.gov/).

Co-culture experiments of MCF7 and patient-derived MSCs with or without A83-01 or Galunisertib (LY2157299) (Selleck Chemicals, Houston, TX) treatments were performed by initially treating MSCs with 7.5 nM of A83-01 or 2 μM Galunisertib for 24 hr. After removal of A83-01, MSCs were treated with CellTracker CMFDA Dye (Invitrogen) for 1hr. After washing cells were then mixed with MCF-7 cells stably expressing mCherry and/or pretreated with CellTracker CMPTX Dye. After co-culture, cells were dissociated by trypsin treatment, washed, suspended in PBS with 5% BSA sorted on BD FACSAria (UVM Larner College of Medicine Harry Hood Bassett Flow Cytometry and Cell Sorting (FCCS) facility). Isolated cells were then processed for RNASeq, pPCR or downstream analysis.

### RNA-seq and Laser Capture Microdissection (LCM) of FFPE tissues

FFPE blocks were obtained from the UVM Surgical Pathology Department after surgical biopsy, excision or mastectomy. All specimen blocks were de-identified and sectioned sequentially for Hematoxylin-Eosin (H&E) staining, Laser Capture Microdissection multiplex or regular immunohistochemistry as previously described [88]. RNA sequencing of archived FFPE tissues was performed using SMART-3Seq, a 3’ tagging strategy specifically designed for degraded RNA directly from FFPE LCM specimens [89]. LCM dissected SMART-3Seq libraries were prepared using the standard protocol for FFPE tissue on Arcturus HS LCM Cap and the individual library SPRI purification option following the established 3Seq protocol [89]. All FFPE LCM dissected libraries were amplified using 19 PCR cycles during indexing to minimize over-amplification of high abundance mRNAs in each library. Libraries were individually analyzed for size distribution on an Agilent Bioanalyzer using High Sensitivity reagent kits to verify average library size of 190 bp and stored at −20°C until samples were sequenced. When all libraries were ready for sequencing, 1 μL of each library was then used to create two library pools used for sequencing and quantified by Qubit 2.0 Fluorometer HS DNA assay. Library pools were sequenced with a 1% PhiX spike-in control library and sequenced using SE75 chemistry (with additional Index read) on an Illumina HiSeq1500 (Vermont Integrated Genomics Resource).

### Single-cell RNA-seq library preparation & sequencing

Patient-derived MSCs were further separated using 10x Genomics Chromium platform to capture and barcode the cells following the manufacturer’s protocol. MSC suspensions were loaded onto 10x Genomics Single Cell fluidics chip and partitioned into single cell containing GEMs which include gel beads coated with specific oligonucleotides containing barcodes to index cells (14 bp) as well as Unique Molecular Identifiers to determine individual transcripts (10 bp UMI). Following cell isolation, reverse transcription was performed, individual cDNAs were amplified, and a library was constructed using the Single Cell 3′ Reagent Kit (v2 chemistry) for each sample. The resulting libraries were sequenced on an Illumina HiSeq 1500 System using PE150 chemistry at the Vermont Integrative Genomics Resource Massively Parallel Sequencing Facility in the Larner College of Medicine at the University of Vermont. After samples were demultiplexed, individual fastq files were subjected to barcode processing and UMI counting using Cell Ranger v2.1.0 (https://support.10xgenomics.com). Each library was processed by alignment to the human reference genome (hg38) using count function to produce a gene count matrix for each cell/library. Barcodes and unique molecular identifiers associated with individual cell assignments were matched with aligned reads were subjected to correction and filtering using an estimation of 3000 recovered cells (—expect-cells 3000). The resulting count matrices for each sample/experiemnt were then concatenated into one matrix using the CellRanger aggregation function. The libraries were normalized to the equivalent read depth and the normalized count matrix was then imported into Seurat [90] for further processing. Poor quality cells were filtered by excluding multiplets or other outliers based on the number of genes detected, the sum of UMI counts and the proportion of UMI counts for mitochondrial genes and normalized the sum of UMI counts for each cell to the median counts of all cells. Batch correction between patient samples and normal MSCs was performed by using the R package scran [91] and Seurat [92]. The first 30 principal components were used to cluster the cells into subpopulations through a graph-based unsupervised clustering approach implemented in Seurat (the “FindClusters” function, resolution = 0.4). Following clustering, the same principal components were used to project the clustered cells onto a two-dimensional (2D) map for visualization by means of t-distributed stochastic neighbor embedding (t-SNE).

## Supporting information

S1 Fig Cell surface marker expression by FACS analysis. Whole tumor samples from breast cancer patients were digested into single cell suspensions and selected for CD44 negative population (MACS). A) The resulting cells were analyzed for CD90 and CD45 expression. A small population (0.3–0.8%) of CD45+CD90+ MSC cells were identified in patient samples (representative experiment). B) CD90+ cells were counted in patient samples and plotted as mean number of 3 replicate counts.
(TIFF)

S1 Table Patient derived tumor and stromal cell samples used for study. Tumor type and library construction information for RNAseq experiments. Library sequencing depth and aligned reads for individual samples used in the study.
(XLSX)

S2 Table Raw gene counts, gene expression values and single cell matrix files used in the study. Quality control analysis of fastq raw data was performed using FastQC [93]. Reads were aligned to reference genome (hg38) using STAR, and reads were quantified using HTSeq-counts with Gencode annotation v38 and raw counts provided in **Table 2a**. Differential expression analysis was performed with DESeq. For differential gene expression analyses, the cutoff for significant fold change was >1.5, adjusted p-value <0.05 and provided in **Table 2b**. *Single cell data*: After samples were demultiplexed, individual fastq files were subjected to barcode processing and UMI counting using Cell Ranger v2.1.0 (https://support.10xgenomics.com). Each individual library was processed using cellranger count function to generate a gene-barcode matrix for each library and reads aligned to the human reference genome (hg38). Cell barcodes and UMIs associated with the aligned reads were subjected to correction and filtering using an estimation of 3000 recovered cells (—expect-cells 3000). The resulting gene-cell UMI count matrices for each sample were then concatenated into one matrix using the “cellranger aggr” pipeline and files are provided as compressed files in **Table 2c.**
(ZIP)

S3 Table Ontology analysis of gene expression data. Gene expression profiles from heatmap clusters highlighting expression groups with similar expression patterns were merged and Gene Ontology (GO) annotation analyses of gene sets were performed using GSEA/MSIgDB (Broad). GO Term enrichment was considered significant for all terms with P<0.05. GO terms were consolidated using REVIGO package on R-Studio. Statistically significant results for each cluster are provided in Table 3b.
(ZIP)

S1 Data (TSV)

S2 Data (TSV)

S3 Data (MTX)
