# Supplementary figures and images for: Identification of molecularly unique tumor-associated mesenchymal stromal cells in breast cancer patients
Source: PLoS One. 2023 Mar 20;18(3):e0282473. doi: 10.1371/journal.pone.0282473 (PMC10027225; doi:10.1371/journal.pone.0282473)

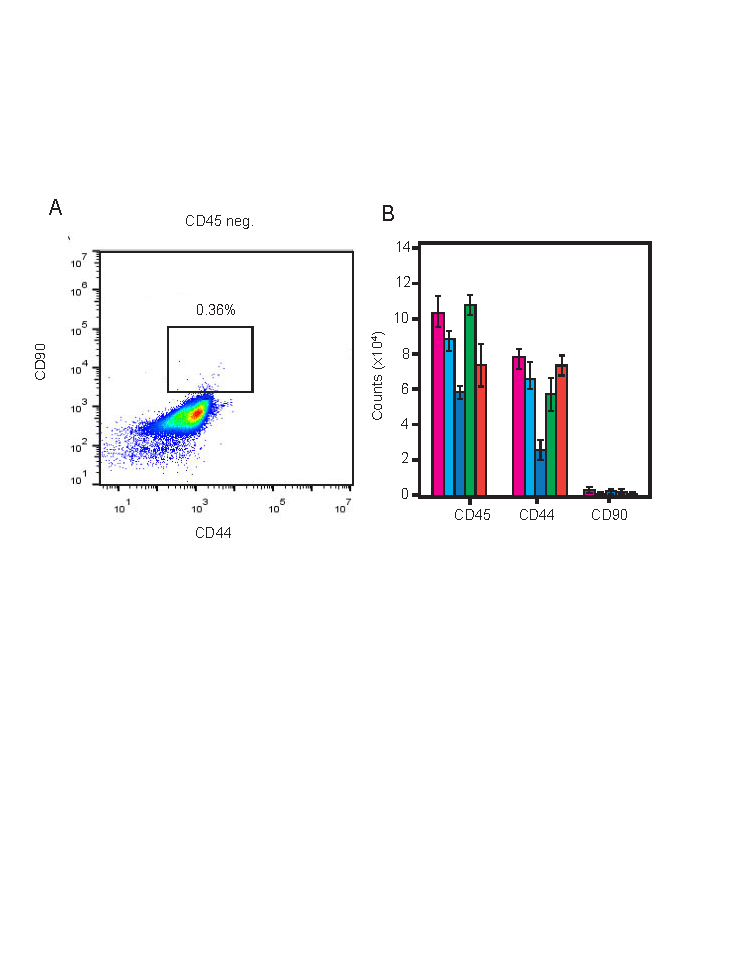

Supplement: S1 Fig — Whole tumor samples from breast cancer patients were digested into single cell suspensions and selected for CD44 negative population (MACS). A) The resulting cells were analyzed for CD90 and CD45 expression. A small population (0.3–0.8%) of CD45+CD90+ MSC cells were identified in patient samples (representative experiment). B) CD90+ cells were counted in patient samples and plotted as mean number of 3 replicate counts. (TIFF) [file pone.0282473.s001.tiff]
